# Physiotherapy Approach to an Internal Capsule Infarct With Upper Motor Neuron Facial Nerve Palsy: A Case Report

**DOI:** 10.7759/cureus.55337

**Published:** 2024-03-01

**Authors:** Achal Mantri, Pallavi Harjpal, Nitika Chavan

**Affiliations:** 1 Department of Neuro-Physiotherapy, Ravi Nair Physiotherapy College, Datta Meghe Institute of Higher Education and Research, Wardha, IND

**Keywords:** post-stroke, rood’s approach, slurred speech, facial palsy, anterior choroidal artery, physiotherapy, rehabilitation, posterior limb, stroke, internal capsule

## Abstract

The internal capsule (IC) is a vital brain structure housing descending and ascending fiber tracts, with traditional assumptions about the corticobulbar and corticospinal tracts descending through the genu and anterior third of the posterior limb of internal capsule (PLIC), respectively. However, observations of IC infarctions reveal that symptoms often deviate from the expected fiber pattern, prompting a deeper exploration of these complexities. The posterior limb of the IC receives its blood supply from the lenticulostriate branches of the middle cerebral artery and the anterior choroidal artery (AChA). AChA infarctions present a diverse array of symptoms beyond the classic triad, reflecting the intricate vascular supply and lesion patterns within this region. We present a case of a 74-year-old male farmer with right-hand dominance, who experienced a fall resulting in head and right lower limb injuries. Subsequently, he developed weakness in his left upper and lower limbs, facial deviation, slurred speech, and swelling in the right lower limb. Following these symptoms, his family promptly brought him to the hospital on November 30, 2023. Extensive investigations, including magnetic resonance imaging (MRI), revealed a hyper-acute infarct in the posterior limb of the left IC. The patient was admitted to the intensive care unit (ICU) for three days and later shifted to the neurology ward where medical management was commenced, including physiotherapy protocol that was started on December 2, 2023. Physiotherapy interventions were designed to address the patient's weakness, altered sensation, and diminished reflexes. Therapeutic goals focused on preventing complications, improving posture, enhancing range of motion (ROM), and mitigating breathing difficulties and mobility issues. The physiotherapy aimed to enhance the patient's overall physical and mental well-being, emphasizing independence and improved quality of life. Regular assessments and adjustments to the therapeutic interventions were made based on the patient's progress. This case underscores the importance of tailored physiotherapy interventions in addressing the diverse manifestations of IC infarctions, contributing to a comprehensive understanding of rehabilitation strategies in neurologically compromised individuals.

## Introduction

The internal capsule (IC) is a brain structure composed of descending and ascending fiber tracts. The corticobulbar tract and the corticospinal tract are assumed to descend independently through the genu as well as the anterior third of the posterior limb of internal capsule (PLIC). Corticospinal tracts in the PLIC are organized across a long axis, thus hand fibers are anteromedial to the foot fibers [1]. Damage to these pathways can disrupt communication between different regions of the brain and lead to motor deficiency, a key player in motor coordination and control with implications for neurological disorders [2]. In our experience, some people have infarction and their symptoms do not always correspond to the traditional fiber pattern [1]. The PLIC is supplied by lenticulostriate branches of the middle cerebral artery (MCA) and the anterior choroidal artery (AChA). The AChA is a thin artery that branches through the outermost part of the internal carotid artery (ICA), 2-5 mm proximal to the skull division. The medial temporal lobe, optic radiations, lateral thalamus, caudate nucleus tail, lateral geniculate body, and medial part of the pallidum are all found in the posterior limb of the IC [3].

The normal, complete AChA condition contains hemi-paresis, hemi-anesthesia, and homonymous hemianopsia; however, the severity of the symptoms varies greatly [3]. These result in a wide range of symptoms related to the brain, commonly with a continuous or intermittent pattern [4]. The overall prevalence of AChA infarction was modest (2.9%) prior to the increasing popularity of magnetic resonance imaging (MRI) in stroke therapy [5]. The probability of isolated AChA infarction was found to be 8.1% in a prospective stroke registry that employed a rigorous MRI technique encompassing at least diffusion-weighted imaging, fluid-attenuated inversion recovery, gradient echo, and time-of-flight images. AChA infarction encompasses various mechanisms, including small and large artery disorders [6]. The basic aim of rehabilitation and physiotherapy is to increase an individual's functioning abilities, independence, and overall quality of life. Physiotherapy is the most prevalent type of recovery after a stroke. Physiotherapists help stroke patients regain the ability to walk, dress, and wash by improving their strength, coordination, and balance. Therapy may involve exercises, stretching, and range of motion (ROM) acts, as well as teaching through assistance equipment such as walkers or canes [7]. In this study, neuro-muscular electrical stimulation and constraint-induced mobility training (CIMT) are used as interventions for recovery after stroke as the patient complained about the deviation of the angle of the mouth and reduced mobility of the upper and lower limbs of the left side.

## Case presentation

A male patient aged 74, whose occupation is farming and who predominantly uses his right hand, presented to the medicine or clinical outpatient department with a history of a fall at his farm while working. The fall resulted in injuries to his head and right lower limb, causing pain. However, he initially ignored the complaints. Subsequently, he began experiencing weakness in the unilateral extremities on the left side, along with deviation in the angle of the mouth, slurred speech, and swelling in the right lower limb. Concerned about these symptoms, his family promptly brought him to the hospital on November 30, 2023. Investigations, including X-ray and MRI, were done to assess the extent of his injuries and determine the underlying cause of his neurological symptoms. Following the interventions, the patient was advised to undergo physiotherapy from December 2, 2023. This treatment plan aimed at addressing the physical effects and assisting in the rehabilitation of any neurological deficit that may have occurred. The events are mentioned according to the dates in the timeline in Table 1.

**Table 1:** Timeline

Events	Date
Symptoms occurred	26/11/2023
Admission to the hospital	30/11/2023
Investigations done	30/11/2023
Physiotherapy started	02/12/2023

Investigations

**Figure 1:**
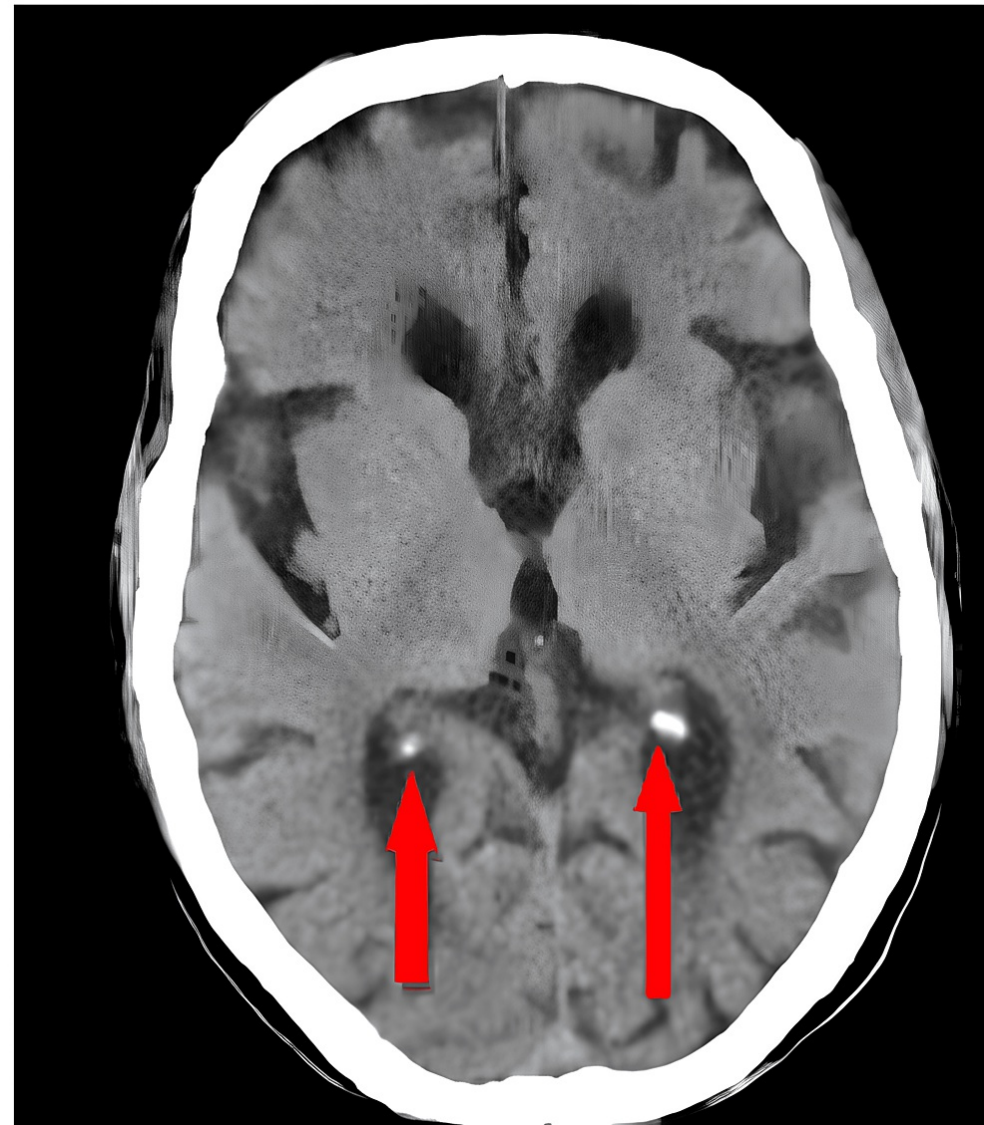
MRI findings of hyper-acute infarct in the posterior limb of the left internal capsule (red arrows)

MRI revealed a hyper-acute infarct in the posterior limb of the left IC, a chronic infarct in the right thalamus and left corona radiate, and age-related atrophic changes with small vessel ischemic disease, as shown in Figure 1 in hyperdense color. Accordingly, the patient was admitted to the medicine intensive care unit (ICU), where medications like ecosprin tablet 75 mg once daily and clopitab tablet 75 mg once daily were given along with physiotherapy treatment. As per the findings, the physiotherapy protocol was planned, and it included proper positioning, upper limb and lower limb strength training exercises, and bed mobility exercises in order to improve the overall physical and mental health of the patient while also increasing the confidence of the patient. The MRI findings are shown in Figure 1.

After obtaining consent from the patient, physiotherapy assessment and management were started. The patient was well-oriented and afebrile. On observation, the patient was seen in a supine lying position with head-end elevated to 30 degrees, and on six liters of oxygen via facemask; Foley's catheter was attached. On examination, the Glasgow Coma Scale score was E4, V5, M6 (15/15). The patient's vitals were: heart rate 100 breaths/min, oxygen saturation 100%, and respiratory rate 20 beats/min. All the sensations were intact. Reflexes and manual muscle testing (MMT) are shown in Table 2. The patient was functionally able to sit with minimum assistance. The ICU mobility scale score was one (sitting in bed) [8]. Reflexes were taken, as shown in Table 2. As the tone was not normal on the left side, a voluntary control grading was taken, as shown in Table 3. As the tone was normal on the right side, MMT was done only on the right side, as shown in Table 4.

**Table 2:** Reflexes (pre-interventional assessment) (+): Diminished reflex; (++): Normal reflex; NA: Not assessable

Reflexes	Right	Left
Biceps reflex	++	+
Triceps reflex	++	+
Brachioradialis reflex	++	+
Patellar reflex	++	+
Plantar reflex	NA (due to cellulitis)	+

**Table 3:** Voluntary control grading (pre-interventional assessment) NA: Not assessable

Voluntary Control Grading		
Muscle test	Right	Left
Shoulder		
Flexor	6/6	1/6
Extensors	6/6	1/6
Elbow		
Flexors	6/6	2/6
Extensors	6/6	2/6
Wrist		
Flexors	6/6	2/6
Extensors	6/6	2/6
Hip		
Flexors	6/6	2/6
Extensors	6/6	2/6
Knee		
Flexors	6/6	1/6
Extensors	6/6	1/6
Ankle		
Dorsiflexors	NA	1/6
Plantarflexors	NA	1/6

**Table 4:** Manual muscle testing (pre-interventional assessment) NA: Not assessable

Manual Muscle Testing (MMT)		
Muscle test	Right	Left
Shoulder		
Flexor	4/5	-
Extensors	4/5	-
Elbow		
Flexors	4/5	-
Extensors	4/5	-
Wrist		
Flexors	4/5	-
Extensors	4/5	-
Hip		
Flexors	4/5	-
Extensors	4/5	-
Knee		
Flexors	2/5	-
Extensors	2/5	-
Ankle		
Dorsiflexors	NA	-
Plantarflexors	NA	-

Therapeutic interventions

**Figure 2:**
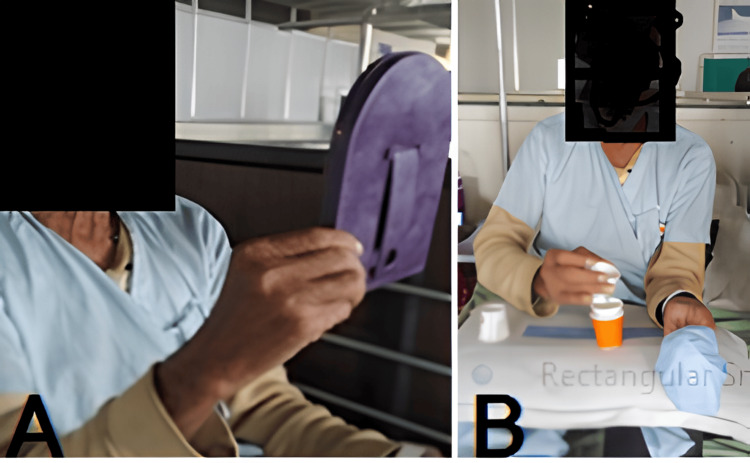
Patient performing (A) facial exercises with mirror feedback and neuromuscular retraining, and (B) constraint-induced movement therapy (A) Mirror feedback is used for muscular retraining of the face; (B) Constraint-induced movement therapy is given to the unaffected extremity and training to the affected

On the day of the assessment, the patient was well-oriented to time, place, and person and was conscious and cooperative. The physiotherapy rehabilitation started in view of the complaints of unilateral extremity weakness of the left side, deviation of the angle of the mouth, and slurred speech. Cellulitis covered with bandaging over the right lower limb was present. The need for and significance of exercising were explained to the patient and his family. To resume activities of daily living with the least amount of difficulties was the aim. Some interventions are shown in Figure 2. Therapeutic interventions are described in Table 5.

**Table 5:** Physiotherapeutic interventions

Problems	Goals	Interventions	Rationale
A lack of understanding of the illness may raise the likelihood of consequences	To prevent complications and risk factors	Patient education	To make the patient independent and improve the quality of life
Lack of ability to maintain proper posture and increased pressure over pressure areas	To improve posture	Positioning	To reduce further complications like bed sores
Reduced tone	To improve tone	Rood’s approach [9]	To improve the tone and improve motor regulation via proprioceptive input
Reduced upper and lower extremities range of motion (ROM)	To improve the ROM of the upper and lower extremity	Active assisted exercises of the upper and lower extremities [10]	Reduced strength
Difficulty in breathing	To reduce the work of breathing	Breathing exercises [11]	Decrease the occurrence and severity of secondary problems
Difficulty in mobility	To reduce secondary complications that occur due to prolonged immobility	Bed mobility (supine-side lying-sitting)	Decrease the occurrence and severity of secondary problems like bed sores and pressure ulcers
Deviation in the angle of the mouth	To reduce the deviation in the angle of the mouth	Facial exercises with mirror feedback and neuromuscular retraining [12]	To improve muscle strength, coordination, and control of facial expressions
The deviated angle of the mouth	To reduce the deviated angle of the mouth	Electrical stimulation techniques such as neuromuscular electrical stimulation (NMES) [13]	To stimulate facial muscles and enhance motor recovery
Limited mobility on the affected side due to a neurological injury or condition	Enhance the individual's ability to move the affected limb and engage in mobility-related tasks	Constraint-induced movement therapy (CIMT) [14]	To encourage the use of the affected limb, promoting neural plasticity and recovery
Slurred speech	Enhance speech	Speech and swallowing therapy [15]	To improve clarity in speech
Unable to do activities of daily living	Enhance the ability to perform activities of daily living	Task-specific training [16]	To improve the mobility and ability to perform the task of daily routine
Agitation due to prolonged immobility and poor health	Enhance health by motivating the patient and improving confidence	Cognitive behavior therapy [17]	To limit agitation and improve the motivation towards physiotherapy for a better lifestyle

As the patient gained voluntary control grading of more than five, so MMT was performed on the same side as shown in Tables 6-7.

**Table 6:** Reflexes (post-interventional assessment) After one month follow-up (+): Diminished reflex; (++): Normal reflex; NA: Not assessable

Reflexes	Right	Left
Biceps reflex	++	++
Triceps reflex	++	++
Brachioradialis reflex	++	++
Patellar reflex	++	++
Plantar reflex	NA (not assessable due to cellulitis)	++

**Table 7:** Manual muscle testing (post-interventional assessment) After one month follow-up NA: Not assessable

Manual Muscle Testing (MMT)		
Muscle test	Right	Left
Shoulder		
Flexor	4/5	4/5
Extensors	4/5	4/5
Elbow		
Flexors	4/5	4/5
Extensors	4/5	4/5
Wrist		
Flexors	4/5	4/5
Extensors	4/5	4/5
Hip		
Flexors	4/5	4/5
Extensors	4/5	4/5
Knee		
Flexors	4/5	3/5
Extensors	4/5	3/5
Ankle		
Dorsiflexors	NA (not assessable due to cellulitis)	3/5
Plantarflexors	NA (not assessable due to cellulitis)	3/5

## Discussion

The studies conducted by Qian et al., Derflinger et al., Högg et al., and Myint et al. provide valuable insights into various aspects of neurological and rehabilitative research [2,4,10,11]. Qian et al. focused on the anatomical positioning of fibers in the PLIC [2]. According to their findings, hand fibers are situated laterally to foot fibers in the short axis while face fibers reside in the remedial region [4]. After obtaining the proper consent of the patient, the whole physiotherapeutic intervention was planned and given accordingly. To educate the patient and his family about the condition and how exactly physiotherapy would help him improve his health, patient education was done on a priority basis. To improve tone, Rood’s approach on the left upper and lower limbs was given. As the patient complained of unilateral extremity weakness on the left side, active assisted ROM for the upper and lower extremities was given to improve strength [10]. To reduce tonal abnormality, Rood's approach was given. In Rood's technique, sensory input is utilized to define particular motor actions, and phases of development are followed, that is, at lower to higher stages sensory-motor actions are practiced when comprehension is attained. To reduce the chances of developing bed sores or pressure ulcers, positioning was described to the patient with proper pillow placement. In such conditions where prolonged bed immobility is present, bed mobility becomes very important. For that, supine, side lying, and sitting exercises were given to the patient. For difficulty in speech, speech therapy and swallowing therapy were given to the patient. In order to reduce breathing difficulties, exercises were given to the patient [11,15].

The deviated angle of the mouth was one of the chief complaints of the patient for which facial exercises with mirror feedback were given. The patient was asked to perform the movement of facial muscles while observing himself in the mirror so that he could get feedback at the same time, which would help him reduce the deviation of the mouth [12]. Because of genetic, pathophysiologic, sociodemographic, and clinical aspects, post-stroke motor recovery is complicated. Neuromuscular electrical stimulation (NMES) is one of the therapeutic methods designed to attempt to stimulate motor recovery [17]. Because normal electrical excitability typically survives in lower motor neurons and their innervated muscles following stroke, NMES can be utilized to activate the neuromuscular activity of the paretic limbs [18]. CIMT was developed to handle upper-extremity issues after a stroke and remains the widely investigated strategy for managing those with stroke in recent decades [19]. As the patient progresses through physiotherapy, it is anticipated that the tailored interventions will contribute to increased independence, improved quality of life, and better functional abilities [20].

Medically, paresis manifests as muscular weakness, decreased activation speed, and failure to create physiologically effective movements of the affected limb [21]. Lang and colleagues investigated the relative strength of the correlations among particular upper limb deficits and function, concluding that paresis was the most significant contributor to function loss [21]. The upper limb's assortment of paresis, loss of fractionated motions, flexor hypertonia, and somatosensory anomalies frequently appear as difficulties extending the elbow and opening the hand in a functional way, seriously restricting the functional workplace. CIMT and modified forms of CIMT (mCIMT) are now regarded as the most efficient rehabilitation protocols for improving upper limb outcomes. Although various systematic examinations have been undertaken in the past, there has been no current meta-analysis of randomized controlled trials (RCTs) of mCIMT that include extensive evaluations of potential impact enhancers and small-study impacts [22].

## Conclusions

In this case, the physiotherapy protocol was aimed at addressing a spectrum of challenges, including weakness in the left upper and lower limbs, facial deviation, slurred speech, and swelling in the right lower limb. Therapeutic interventions were meticulously tailored to optimize the patient's overall physical and mental well-being, emphasizing precise positioning, targeted strength training, and enhancing bed mobility. This case introduces novel therapeutic strategies that significantly contribute to the existing literature, offering tailored interventions that surpass conventional therapies and potentially broaden the array of treatment options for similar cases in the future. It underscores the significance of a comprehensive, patient-centered approach in managing IC infarction, acknowledging the diverse array of symptoms and emphasizing the necessity for personalized rehabilitation strategies to achieve optimal recovery.

## References

[1] Qian C, Tan F (2017). Internal capsule: the homunculus distribution in the posterior limb. Brain Behav.

[2] Ogut E, Armagan K, Tufekci D (2023). The Guillain-Mollaret triangle: a key player in motor coordination and control with implications for neurological disorders. Neurosurg Rev.

[3] Chausson N, Joux J, Saint-Vil M (2014). Infarction in the anterior choroidal artery territory: clinical progression and prognosis factors. J Stroke Cerebrovasc Dis.

[4] Takahashi S, Ishii K, Matsumoto K, Higano S, Ishibashi T, Suzuki M, Sakamoto K (1994). The anterior choroidal artery syndrome. Neuroradiology.

[5] Derflinger S, Fiebach JB, Böttger S, Haberl RL, Audebert HJ (2015). The progressive course of neurological symptoms in anterior choroidal artery infarcts. Int J Stroke.

[6] Paroni Sterbini GL, Agatiello LM, Stocchi A, Solivetti FM (1987). CT of ischemic infarctions in the territory of the anterior choroidal artery: a review of 28 cases. AJNR Am J Neuroradiol.

[7] Yu J, Xu N, Zhao Y, Yu J (2018). Clinical importance of the anterior choroidal artery: a review of the literature. Int J Med Sci.

[8] Hodgson C, Needham D, Haines K (2014). Feasibility and inter-rater reliability of the ICU mobility scale. Heart Lung.

[9] Chaturvedi P, Kalani A (2023). Motor rehabilitation of aphasic stroke patient: the possibility of Rood's approach. Neural Regen Res.

[10] Bajko Z, Andone S, Maier S (2018). Infarction of the posterior limb of the IC: the first clinical manifestation of lupus. Ro J Neurol.

[11] Shahid J, Kashif A, Shahid MK (2023). A comprehensive review of physical therapy interventions for stroke rehabilitation: impairment-based approaches and functional goals. Brain Sci.

[12] Högg S, Holzgraefe M, Wingendorf I, Mehrholz J, Herrmann C, Obermann M (2019). Upper limb strength training in subacute stroke patients: study protocol of a randomised controlled trial. Trials.

[13] Kang ES, Yook JS, Ha MS (2022). Breathing exercises for improving cognitive function in patients with stroke. J Clin Med.

[14] Robinson MW, Baiungo J (2018). Facial rehabilitation: evaluation and treatment strategies for the patient with facial palsy. Otolaryngol Clin North Am.

[15] Mäkelä E, Venesvirta H, Ilves M (2019). Facial muscle reanimation by transcutaneous electrical stimulation for peripheral facial nerve palsy. J Med Eng Technol.

[16] Myint JM, Yuen GF, Yu TK (2008). A study of constraint-induced movement therapy in subacute stroke patients in Hong Kong. Clin Rehabil.

[17] Wilkinson JM, Codipilly DC, Wilfahrt RP (2021). Dysphagia: evaluation and collaborative management. Am Fam Physician.

[18] Knutson JS, Fu MJ, Sheffler LR, Chae J (2015). Neuromuscular electrical stimulation for motor restoration in hemiplegia. Phys Med Rehabil Clin N Am.

[19] Kim KH, Jang SH (2021). Effects of task-specific training after cognitive sensorimotor exercise on proprioception, spasticity, and gait speed in stroke patients: a randomized controlled study. Medicina (Kaunas).

[20] Rost NS, Brodtmann A, Pase MP (2022). Post-stroke cognitive impairment and dementia. Circ Res.

[21] Kwakkel G, Veerbeek JM, van Wegen EE, Wolf SL (2015). Constraint-induced movement therapy after stroke. Lancet Neurol.

[22] Wang D, Xiang J, He Y, Yuan M, Dong L, Ye Z, Mao W (2022). The mechanism and clinical application of constraint-induced movement therapy in stroke rehabilitation. Front Behav Neurosci.

